# Low temperature scalable synthetic approach enabling high bifunctional electrocatalytic performance of NiCo_2_S_4_ and CuCo_2_S_4_ thiospinels†

**DOI:** 10.1039/d1ra02309h

**Published:** 2021-09-24

**Authors:** Ginena Bildard Shombe, Shumaila Razzaque, Malik Dilshad Khan, Tebello Nyokong, Philani Mashazi, Jonghyun Choi, Sanket Bhoyate, Ram K. Gupta, Neerish Revaprasadu

**Affiliations:** Department of Chemistry, University of Zululand Private Bag X1001 KwaDlangezwa 3880 South Africa RevaprasaduN@unizulu.ac.za malikdilshad@hotmail.com; Chemistry Department, University of Dar es Salaam P.O. Box 35061 Dar es Salaam Tanzania; Key Laboratory of Material Chemistry for Energy Conversion and Storage, Ministry of Education, Hubei Key Laboratory of Material Chemistry and Service Failure, School of Chemistry and Chemical Engineering, Huazhong University of Science and Technology Luoyu Road No. 1037 Wuhan China; Institute of Physical Chemistry, Polish Academy of Sciences Kasprzaka 44/52 01-224 Warsaw Poland; Institute for Nanotechnology Innovation, Rhodes University P.O. Box 94 Makhanda 6140 South Africa; Department of Chemistry, Rhodes University P. O. Box 94 Makhanda 6140 South Africa; Department of Chemistry, Pittsburg State University Pittsburg KS 66762 USA

## Abstract

Ternary metal sulfides are currently in the spotlight as promising electroactive materials for high-performance energy storage and/or conversion technologies. Extensive research on metal sulfides has indicated that, amongst other factors, the electrochemical properties of the materials are strongly influenced by the synthetic protocol employed. Herein, we report the electrochemical performance of uncapped NiCo_2_S_4_ and CuCo_2_S_4_ ternary systems prepared *via* solventless thermolysis of the respective metal ethyl xanthate precursors at 200 and 300 °C. The structural, morphological and compositional properties of the synthesized nanoparticles were examined by powder X-ray diffraction (p-XRD), transmission electron microscopy (TEM), high-resolution TEM, scanning electron microscopy (SEM), X-ray photoelectron spectroscopy (XPS) and energy-dispersive X-ray spectroscopy (EDX) techniques. Electrochemical studies indicate that NiCo_2_S_4_ nanoparticles synthesized at 300 °C exhibit superior energy storage characteristics with a high specific capacitance of *ca*. 2650 F g^−1^ at 1 mV s^−1^, as compared to CuCo_2_S_4_ nanoparticles, which showcased a specific capacitance of *ca*. 1700 F g^−1^ at the same scan rate. At a current density of 0.5 A g^−1^, NiCo_2_S_4_ and CuCo_2_S_4_ nanoparticles displayed specific capacitances of 1201 and 475 F g^−1^, respectively. In contrast, CuCo_2_S_4_ nanoparticles presented a higher electrocatalytic activity with low overpotentials of 269 mV for oxygen evolution reaction (OER), and 224 mV for the hydrogen evolution reaction (HER), at 10 mA cm^−2^. The stability of the catalysts was examined for 2000 cycles in which a negligible change in both OER and HER activities was observed.

## Abstract 2

A scalable solventless approach is employed to prepare NiCo_2_S_4_ and CuCo_2_S_4_ with bare surface for enhanced supercapacitance and water splitting. The particles exhibit good energy storage and electrocatalytic activity as well as stability.

## Graphical abstract

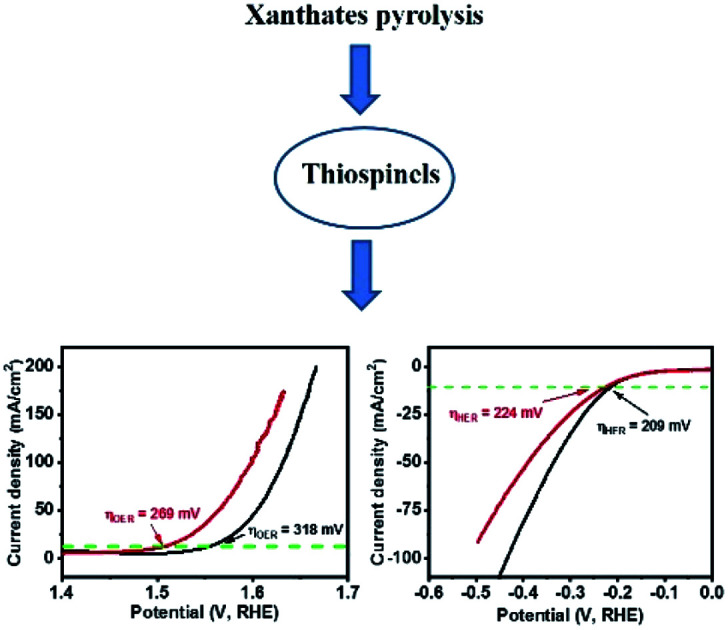

## 1. Introduction

Designing energy storage devices and conversion technologies based on clean and renewable energy is critical for sustainable energy development. Supercapacitors have demonstrated high functional capability in terms of power density, lifecycle, and charge–discharge rate and have therefore received great attention as efficient energy storage devices.^1^ Responding to the projected high energy consumptions, recent studies have mainly focused on improving device energy density and rate capability.^2^ The hydrogen production *via* the electrochemical splitting of water is another intensively investigated strategy of renewable energy storage and/or conversion.^3^ While the approach presents a simple way of producing high purity hydrogen, its practical application is impeded by the large overpotential involved.^3,4^ To attain faster reaction kinetics and scale-up hydrogen production, the process requires the use of earth-abundant, cost-effective and highly efficient electrocatalysts.^5^ On this account, numerous electroactive materials have been investigated for both energy storage and energy conversion applications. Transition metal nitrides,^6^ transition metal dichalcogenides,^7^ hybrids of metals and metal phosphides/metal nitrides,^8^ carbon nanomaterials, their doped derivatives, and composites,^9^ and ternary thiospinels,^1*b*,10^ are examples of advanced materials which have demonstrated remarkable performance.

NiCo_2_S is a normal thiospinel with remarkable electrochemical performance.^1*b*,11^ This superior performance is believed to be due to the high conductivity of the material, which is attributed to its metallic character.^11^ Since the first report by Chen *et al.*,^12^ a large number of studies on the use of NiCo_2_S_4_ in supercapacitors,^13^ batteries,^14^ dye-sensitized solar cells,^15^ oxygen reduction/evolution reactions,^16^ and hydrogen generation reactions^17^ have been carried out. Recently, CuCo_2_S_4_, another member of the ternary thiospinels, has been reported for its potential in energy storage/conversion applications.^18^ Similar to NiCo_2_S_4_, CuCo_2_S_4_, has a normal thiospinel crystal structure with copper and cobalt ions occupying the tetrahedral and octahedral sites, respectively.^19^ It is paramagnetic and metallic, with unique electronic properties. For instance, it exhibits a low resistivity (10^−4^ Ω), and s-wave superconductivity at low temperature.^20^ Specifically, CuCo_2_S_4_ has mainly been studied as an electrode material for lithium-ion batteries,^18*c*^ sodium-ion batteries,^18*b*^ and supercapacitors.^18*e*^ Limited studies on the electrocatalytic activity of CuCo_2_S_4_ in oxygen reduction/evolution reactions,^18*d*^ and hydrogen generation reactions^18*a*^ are known. Both bulk and nanostructured NiCo_2_S_4_/CuCo_2_S_4_ based materials have been studied. It has been shown that, nano-engineering of the electrodes/electrocatalysts enhances the electrochemical performance of the materials.^1*b*,21^

The synthetic protocol employed in nanomaterials preparation is among the key factors determining the final structure and resultant properties of the materials.^1*b*,22^ Years of extensive research on nanoscience and nanotechnology has provided us with numerous synthetic approaches, with solution-based techniques dominating the field.^23^ A characteristic feature of nanomaterials synthesized in solution is the presence of surface adsorbed ligands which provide particle stabilization *via* electrostatic or steric repulsive interactions. Long-chain amines, carboxylates, thiols, and phosphines are some of the commonly employed ligands.^24^ While capping ligands are indispensable during nanoparticles synthesis, studies have shown that the presence of these ligands on the surface of a nanoparticle may, in some cases, act as impurities, blocking the particle's active sites and diminish the material's end application, particularly catalysis. For example, Ung *et al.*^25^ studied the effect of various amine and carboxylate ligands on the electrocatalytic activity of CoP towards hydrogen generation in which the uncapped CoP nanocrystals demonstrated higher activity than the capped nanocrystals. Lu *et al.*^26^ examined the electrocatalytic activity of Ag nanorods for oxygen reduction reaction in which the surfactant-free nanorods displayed higher activity than the nanorods capped with polyvinylpyrrolidone. In another study, Henckel *et al.*^27^ investigated the role of dodecylamine, as a surfactant, on the hydrogen generation activity of WSe_2_. Removing the ligand from the catalytic sites of the material was observed to improve the kinetics of the reaction. The synthesis of nanomaterials free of capping ligands is therefore crucial for their catalytic performance. Several techniques for the removal of the capping ligands after nanomaterials synthesis have been developed.^26–28^ However, most of these techniques are laborious, expensive, and sometimes alter the shape and stability of the particles.^29^

The melt method is a recently described approach of synthesis which allows the preparation of nanomaterials in the absence of any passivating ligands.^30^ This provides the possibility of synthesizing nanomaterials with abundant active sites for enhanced electrochemical performance. Unlike solution-based techniques, nanomaterials are synthesized *via* solvent-free, surfactant-free thermolytic decomposition of their corresponding molecular precursors.^30,31^ The approach is therefore simple, inexpensive and eco-friendly. Herein, we report the synthesis of uncapped NiCo_2_S_4_ and CuCo_2_S_4_ nanoparticles *via* the melt approach using respective metal ethyl xanthate precursors. The efficiency of the synthesized ternary systems for supercapacitance and water splitting applications has been examined. To the best of our knowledge, this is the first study investigating the electrochemical properties of the two systems synthesized using the melt approach.

## 2. Experimental

### 2.1. Chemicals

Potassium ethyl xanthogente (96%, Sigma-Aldrich), cupric chloride dihydrate (97%, Saarchem), cobalt(ii) acetate tetrahydrate (98%, Saarchem); and nickel(ii) acetate tetrahydrate, chloroform (min 99%), acetone and hexane, purchased from Merck Chemicals. All chemicals were used as received with no further purification.

### 2.2. Instrumentation

Elemental analysis of the complexes was performed on a PerkinElmer automated model 2400 series II CHNS/O analyzer. Thermogravimetric analysis was done from 30 to 600 °C at 10 °C min^−1^ using a PerkinElmer Pyris 6 under N_2_ gas flow. p-XRD analysis was performed using a Bruker AXS D8 diffractometer equipped with a nickel-filtered Cu Kα radiation (*λ* = 1.5418 Å) at 40 kV, 40 mA, at room temperature. The diffraction patterns were recorded in the high angle 2*θ* range of 10–70° at a scan speed of 0.2 s per step and an increment of 0.01314. TEM and HRTEM analyses of the synthesized ternary systems were carried out using a JEOL 1400 TEM and JEOL 2100 HRTEM, respectively. Samples were prepared by placing a drop of the particles dilute solution on Formvar-coated grids (150 mesh) for TEM and holey carbon grids for HRTEM. The samples were allowed to dry completely at room temperature, viewed at accelerating voltages of 120 kV and 200 kV for TEM and HRTEM, respectively. Images were captured digitally using a Mega view III camera; stored and measured using soft imaging systems iTEM software (TEM) and Gatan camera with Gatan software (HRTEM). Analysis of surface morphology of the synthesized particles was carried out using a Zeiss Ultra Plus FEG Scanning Electron Microscope (SEM) equipped with an Oxford detector EDX at 20 kV which uses Aztec software for elemental analysis. Kratos Axis Ultra DLD spectroscopy was used to record the X-ray photoelectron spectroscopy (XPS) and the equipment setup and specification were as previously reported.^32^ Curve fitting and quantitative analysis were conducted using Vision software. NIST XPS database was used for peak assignment for the high-resolution spectra and all binding energies were calibrated against carbon at 284.9 eV.

### 2.3. Synthesis of the precursors

#### 2.3.1. Synthesis of bis(*O*-ethylcarbonodithioato)nickel(ii); complex (1)

In a typical reaction, nickel acetate tetrahydrate (1.2443 g, 5.0 mmol) was dissolved in distilled water (25.0 mL) followed by its drop-wise addition into the aqueous solution of potassium ethyl xanthogenate (1.603 g, 10.0 mmol). The reaction mixture was stirred for 1 hour, after which the formed precipitates were washed with distilled water, dried under vacuum and recrystallized from chloroform.

Elemental analysis for C_6_H_10_O_2_S_4_Ni: calc. C, 23.91%; H, 3.35%; S, 42.51%. Found: C, 23.81%; H, 3.2%; S, 42.17%.

#### 2.3.2. Synthesis of bis(*O*-ethylcarbonodithioato)copper(ii); complex (2)

The synthetic procedure used to prepare complex (1) was followed using copper(ii) chloride dihydrate (0.8524 g, 5 mmol) as the source of copper.

Elemental analysis for C_6_H_10_O_2_S_4_Cu: calc. C, 23.57%; H, 3.30%; S, 41.86%. Found: C, 23.1%; H, 3.14%; S, 41.16%.

#### 2.3.3. Synthesis of bis(*O*-ethylcarbonodithioato)cobalt(ii); complex (3)

Complex (3) was also prepared using a similar synthetic procedure as Section 2.3.2 above, using cobalt(ii) acetate tetrahydrate (1.2454 g, 5 mmol) as the cobalt source.

Elemental analysis for C_6_H_10_O_2_S_4_Co: calc. C, 23.93%; H, 3.35%; S, 42.50%. Found: C, 22.03%; H, 3.33%; S, 41.09%.

### 2.4. The solventless synthesis of NiCo_2_S_4_ and CuCo_2_S_4_ nanoparticles

Stoichiometric quantities of the required complexes (complexes (1) and (3) for NiCo_2_S_4_; and complexes (2) and (3) for CuCo_2_S_4_) were ground together to obtain a homogenized mixture. The mixture was then spread in a ceramic boat and placed at the center of a quartz tube. The sample was heated in the furnace at a specified temperature (200 °C or 300 °C) for one hour under N_2_ flow and then allowed to cool down to room temperature.

### 2.5. Electrochemical studies

Electrochemical characterization of the synthesized materials was performed using Gamry Potentiostat using a three-electrode system. A paste consisting of the synthesized sample (80 wt%), acetylene black (10 wt%), and polyvinylidene difluoride (PVDF, 10 wt%) was prepared using *N*-methyl pyrrolidinone (NMP) as a solvent. This paste was then applied to pre-cleaned and weighted nickel foam. The paste was then dried under a vacuum at 60 °C for 10 hours and used as a working electrode. A graphite rod/platinum wire and saturated calomel electrode (SCE) were used as counter and reference electrodes, respectively. All the experiments for energy storage and electrocatalysis were performed using 3 M and 1 M KOH electrolyte, respectively. Charge storage capacity was measured using cyclic voltammetry (CV) and galvanostatic charge–discharge (CD) at various scan rates and current densities. Electrocatalytic properties of the synthesized electrodes were studied using linear sweep voltammetry (LSV), cyclic voltammetry and chronoamperometry (CA). LSV was performed at a scan rate of 2 mV s^−1^ for both OER and HER measurements.

## 3. Results and discussion

### 3.1. Characterization of the precursors and the nanoparticles

The preparation of complexes (1), (2), and (3) involved a well-known synthetic procedure.^33^ The thermal stability behavior of the complexes was analyzed by TGA and the results are shown in Fig. S1, ESI.† The analysis indicates that all three complexes decompose in a single step to give respective metal sulfide. Specifically, complex (1) shows initial decomposition at 132 °C and complete decomposition occurs at 228 °C. A weight loss of 72% is observed, and the remaining residual weight (28%) agrees well with that of NiS (29%). Complex (2) decomposes at 210 °C with an onset temperature of 77 °C. This yields a 30% residue, which corresponds to the theoretical value calculated for CuS (31%). On the other hand, complex (3) starts decomposing at 96 °C and complete decomposition occurs at 203 °C. A 27% residual weight which correlates with that of CoS (29%), is produced. Generally, all three complexes show complete decomposition at moderate temperatures, which is a characteristic feature of xanthate complexes. Compared to several other precursors, the low decomposition temperature and formation of volatile by-products (COS and an alkene) make xanthates complexes favourable precursors for the solventless synthesis of metal sulfides.^34^

**Fig. 1:**
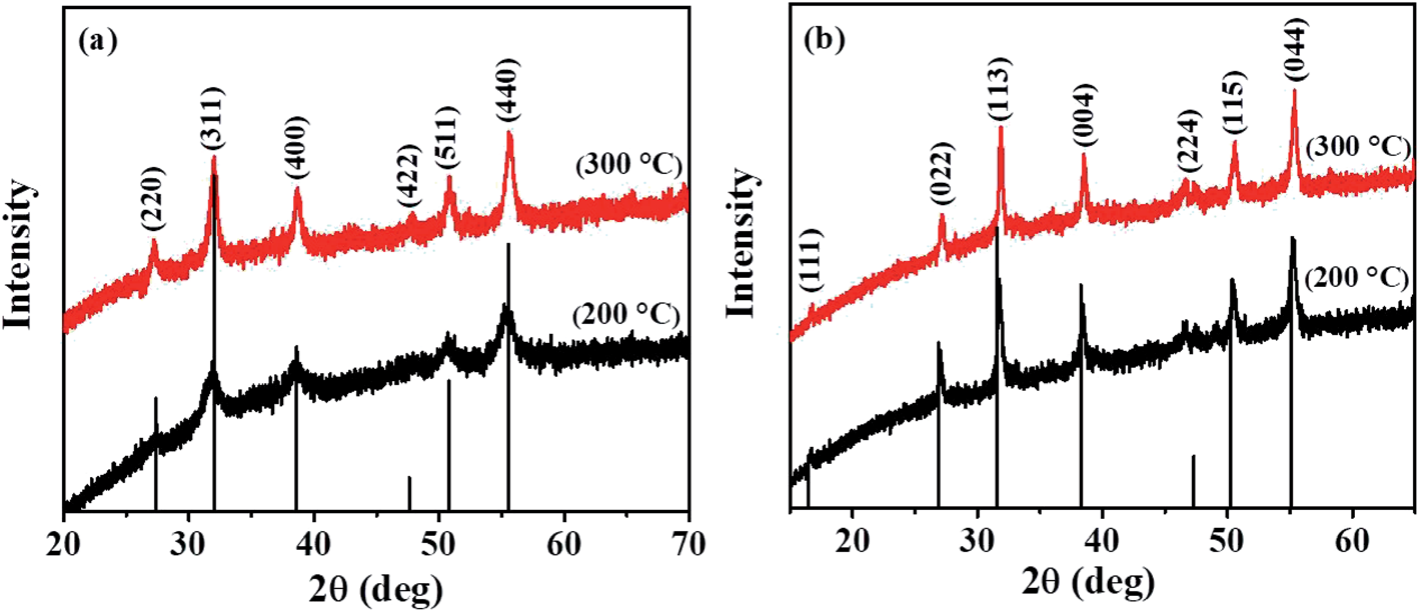
p-XRD patterns of (a) NiCo_2_S_4_ and (b) CuCo_2_S_4_ nanoparticles synthesized at 200 °C and 300 °C.

The two ternary systems were synthesized *via* thermolysis of stoichiometric mixtures of the required complexes at 200 and 300 °C. The solventless pyrolysis of a mixture of complexes (1) and (3) at both 200 and 300 °C produced black residues, which upon p-XRD analysis, were identified as phase-pure cubic NiCo_2_S_4_ (ICDD #: 00-020-0782) (Fig. 1a). The corresponding diffraction peaks are sharp, which denotes the crystallinity of the materials. The relative broad diffraction peaks observed for NiCo_2_S_4_ synthesized at 200 °C are indicative of small-sized particles. The average crystallite sizes were calculated from the Scherrer equation and were found to be 5.3 and 12.8 nm at 200 and 300 °C, respectively. On the other hand, the solventless pyrolysis of a mixture of complexes (2) and (3) at the two temperatures yielded phase-pure cubic CuCo_2_S_4_ (ICDD #: 00-042-1450) (Fig. 1b). The diffraction peaks are also sharp in this case, signifying the synthesis of crystalline CuCo_2_S_4_. The average crystallite sizes of CuCo_2_S_4_ synthesized at 200 and 300 °C were found to be 20.1 and 22.9 nm from the Scherrer equation, respectively.

The lattice parameters of the synthesized materials were calculated from the XRD data using the relation 1/*d*^2^ = (*h*^2^ + *k*^2^ + *l*^2^)/*a*^2^. The values were found to be approximately 9.350 Å and 9.348 Å for NiCo_2_S_4_ nanoparticles synthesized at 200 and 300 °C, respectively, and 9.443 Å for CuCo_2_S_4_ nanoparticles synthesized at 200 and 300 °C, respectively. These values compare well with the theoretical lattice parameters (9.387 Å for NiCo_2_S_4_ and 9.474 Å for CuCo_2_S_4_).

**Fig. 2:**
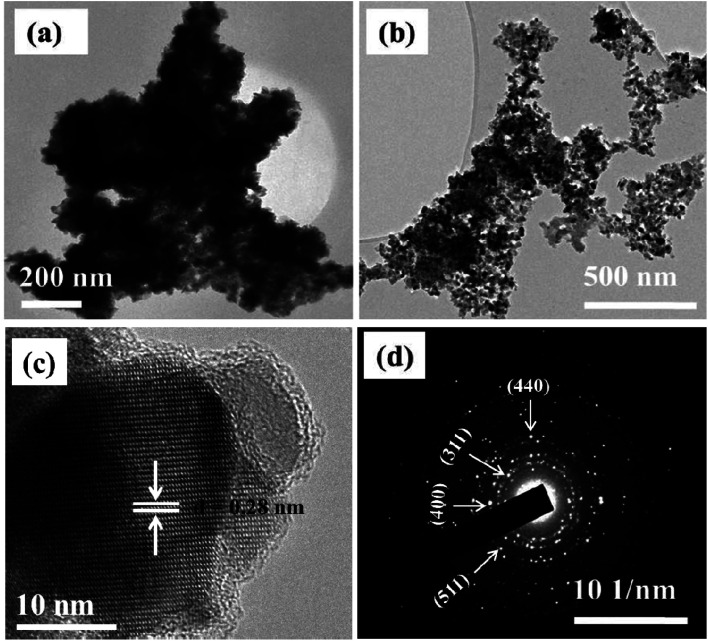
TEM images of NiCo_2_S_4_ nanoparticles synthesized at (a) 200 °C and (b) 300 °C. Corresponding HRTEM (c) and SAED (d) images of the particles synthesized at 300 °C.

Morphological properties of the synthesized materials were examined using TEM/HRTEM and SEM analyses. TEM analysis of NiCo_2_S_4_ prepared at 200 °C reveals the synthesis of highly agglomerated particles with undefined morphology (Fig. 2a). Carrying out the synthesis at a higher reaction temperature of 300 °C produced particles with a more defined morphology and reduced degree of particle–particle agglomeration. Elongated nanoparticles with approximately uniform sizes were obtained (Fig. 2b). The observed average particle size correlates with that calculated from the Debye–Scherrer equation. Due to the high degree of agglomeration, the average size of the particles synthesized at 200 °C could not be estimated from their TEM image. The agglomeration observed at both 200 and 300 °C is mainly due to the absence of a surface passivating agent. In the absence of surfactants, control over size and shape is challenging. The initially formed nano-sized particles have high surface energy and tend to agglomerate to lower their surface energy. Although melt reactions are sometimes characterized as ‘self-capping’, it has been shown that the control over the size and shape of the nanoparticles is mainly achieved with long alkyl chain precursors. For instance, Zhang *et al.*^33*c*^ reported the solventless synthesis of Ag_2_S nanoparticles *via* thermal decomposition of silver xanthate precursors of varying alkyl chain lengths. The size of the particles was observed to decrease with increasing the alkyl chain length of the precursor. O'Brien *et al.*^35^ employed a similar approach to synthesize PbS nanocrystals from various xanthate precursors in which control over the size and shape of the nanocrystals was achieved by increasing precursor chain length. Therefore, the use of short-chain xanthates is thought to be another reason for the observed agglomeration behavior.

The HRTEM image of NiCo_2_S_4_ nanoparticles synthesized at 300 °C shows unidirectional lattice fringes with a *d*-spacing of 0.28 nm, which matches that of the (311) plane of the cubic phase NiCo_2_S_4_ (Fig. 2c). The corresponding selected area electron diffraction (SAED) image shows bright spots, further detailing the crystalline nature of the material. The diffraction rings can be indexed to the (400), (311), (511) and (440) planes of cubic NiCo_2_S_4_ (ICDD #: 00-020-0782) (Fig. 2d).

**Fig. 3:**
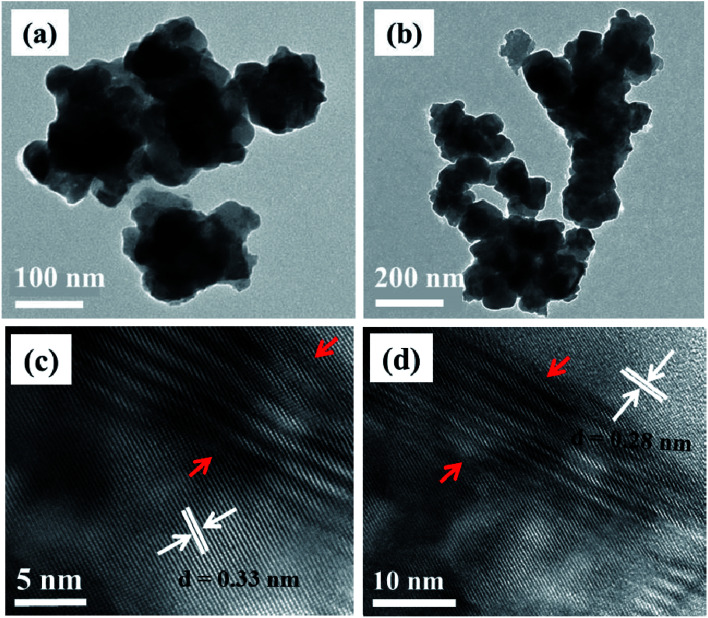
TEM images of CuCo_2_S_4_ nanoparticles synthesized at (a) 200 °C and (b) 300 °C; (c and d) HRTEM images of the particles synthesized at 300 °C.

TEM images of CuCo_2_S_4_ nanoparticles synthesized at both 200 °C and 300 °C are shown to exhibit a sheet-like morphology (Fig. 3a and b). As was the case with NiCo_2_S_4_ nanoparticles, the tendency of particle–particle agglomeration is also observed in this case. The HRTEM images of the particles synthesized at 300 °C show two types of lattice fringes with a *d*-spacing of 0.33 and 0.28 nm which matches that of the (022) and (113) planes of cubic CuCo_2_S_4_, respectively (Fig. 3c and d). The areas shown by red arrows indicate the presence of slightly distorted lattice planes (Fig. 3c). This can be a result of rapid growth, which is a characteristic feature of the melt method.^36^ Rapid growth can cause atoms to be displaced from their original positions, inducing various crystal defects.

**Fig. 4:**
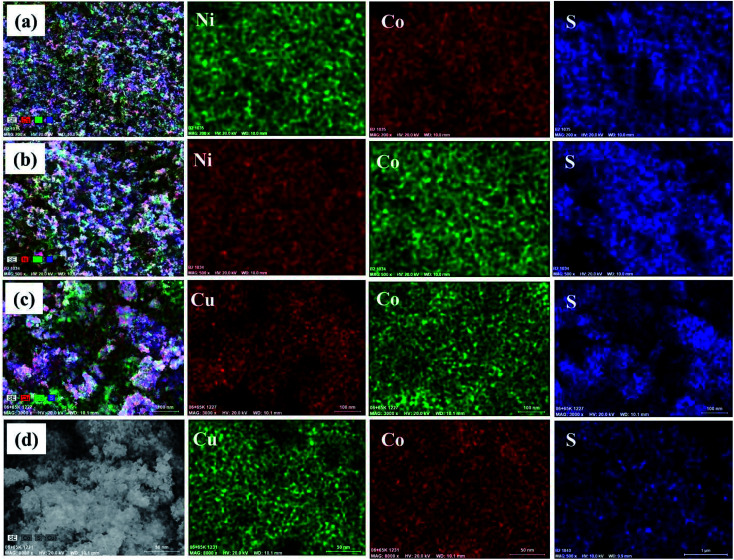
Elemental mapping of NiCo_2_S_4_ nanoparticles synthesized at (a) 200 °C and (b) 300 °C; and CuCo_2_S_4_ nanoparticles synthesized at (c) 200 °C and (d) 300 °C.

The composition of the synthesized materials was examined by Energy Dispersive X-ray Spectroscopy (EDX). The EDX spectra of NiCo_2_S_4_ and CuCo_2_S_4_ nanoparticles synthesized at both 200 and 300 °C indicate the presence of only the desired elements (Ni, Co and S for NiCo_2_S_4_; and Cu, Co and S for CuCo_2_S_4_) (Fig. S2, ESI†). The corresponding quantitative compositions are displayed in Table 1. It can be seen that the obtained experimental compositions, match the expected theoretical compositions with slight deviations (Table 1). Elemental mapping of the samples shows a homogeneous distribution of constituent elements in all the samples (Fig. 4).

**Table 1:** Atomic percent compositions of NiCo_2_S_4_ and CuCo_2_S_4_ nanoparticles

Sample	Pyrolysis temperature (°C)	Atomic% composition				Mole ratio
		Ni	Cu	Co	S	
NiCo_2_S_4_	200	17.10	—	28.67	54.22	1.19 : 2.0 : 3.79
NiCo_2_S_4_	300	17.29	—	27.88	54.84	1.21 : 1.95 : 3.83
CuCo_2_S_4_	200	—	20.05	31.11	48.84	1.4 : 2.17 : 3.4
CuCo_2_S_4_	300	—	20.70	28.84	50.46	1.45 : 2.01 : 3.53

**Fig. 5:**
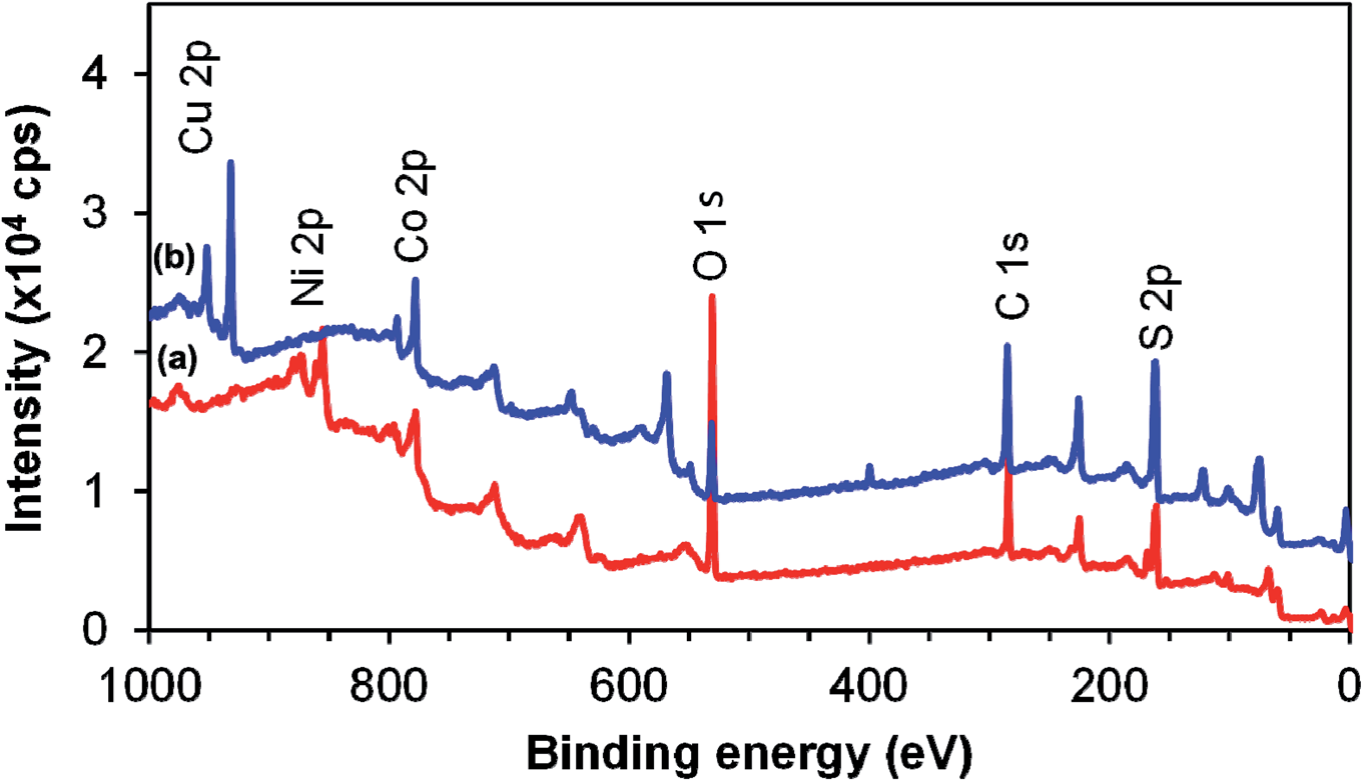
X-ray photoelectron survey spectra of NiCo_2_S_4_ (a) and CuCo_2_S_4_ (b) synthesized at 300 °C.

XPS analysis was carried out to study the surface composition and chemical states of the two ternary systems. NiCo_2_S_4_ and CuCo_2_S_4_ synthesized at 300 °C were used as representative samples. The survey spectra of the two systems are shown in Fig. 5. The presence of Cu 2p peaks at 943 eV for CuCo_2_S_4_ and Ni 2p peaks at 870 eV for NiCo_2_S_4_ together with Co 2p at 790 eV and S 2p at 164 eV confirms that CuCo_2_S_4_ and NiCo_2_S_4_ were successfully synthesized. The O 1s (530 eV) and C 1s (285 eV) peaks resulted from the method used in the preparation of the sample and from the metal ethyl xanthate precursors. A slight stoichiometric variation is evident as the elements nickel, cobalt, and sulfur have the tendency to exist in two mineralogical forms, *i.e.*, NiCo_2_S_4_ or CoNi_2_S_4_. Therefore, with different oxidation states, cobalt can interchangeably occupy tetrahedral or octahedral positions, which may affect the peak intensity and composition.

**Fig. 6:**
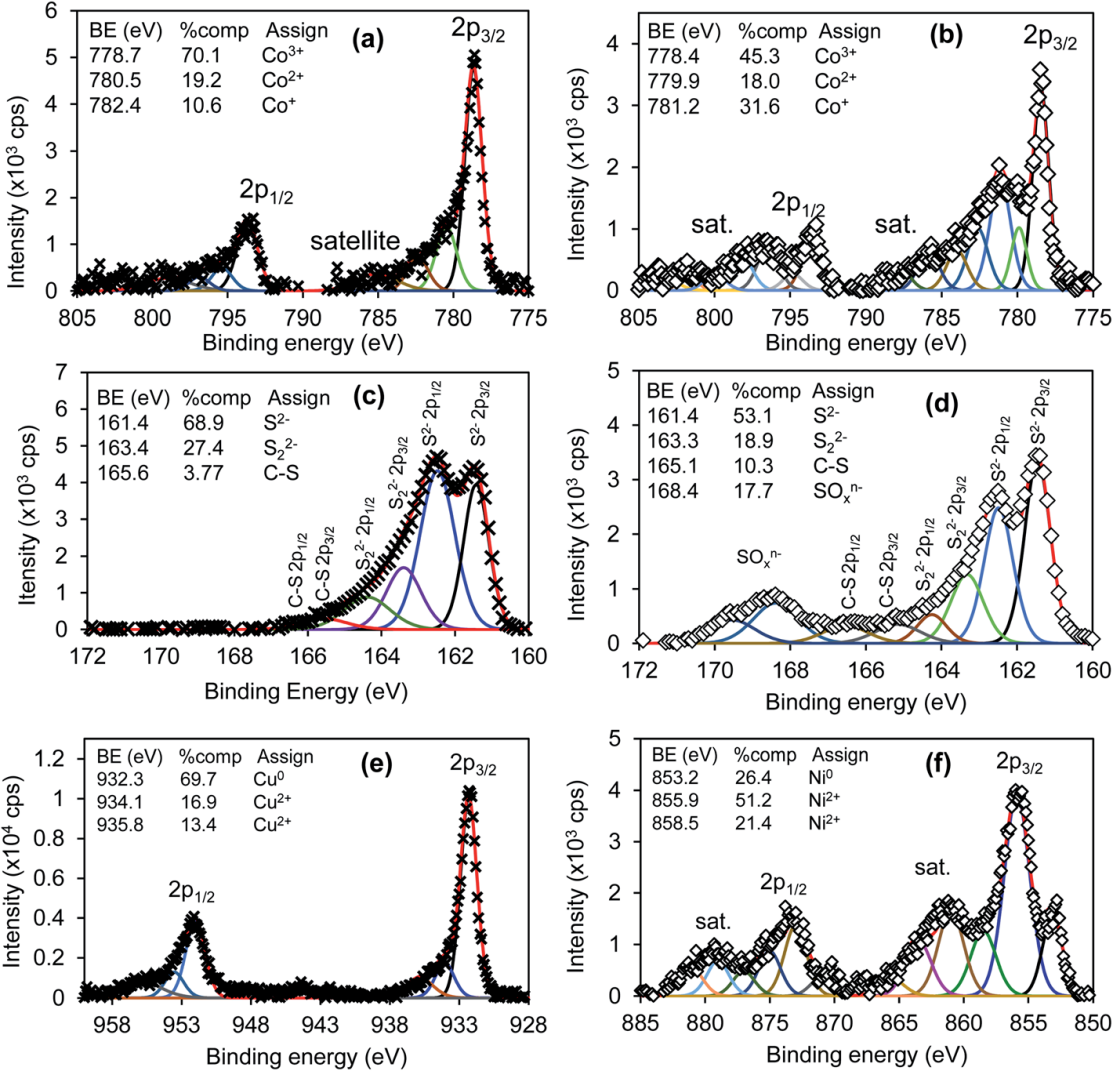
High-resolution spectra of CuCo_2_S_4_ (left column) and NiCo_2_S_4_ (right columns) for (a) and (b) Co 2p; (c) and (d) S 2p; (e) Cu 2p and (f) Ni 2p.

Fig. 6 shows high-resolution spectra of Co 2p for (a) CuCo_2_S_4_ and (b) NiCo_2_S_4_; S 2p for (c) CuCo_2_S_4_ and (d) NiCo_2_S_4_; Cu 2p for CuCo_2_S_4_ (e), and Ni 2p for NiCo_2_S_4_ (f). The Co 2p for both samples showed three oxidation states Co^3+^, Co^2+^ and Co^+^, and the NiCo_2_S_4_ sample showed the presence of strong satellite peaks at high binding energy. The CuCo_2_S_4_ sample exhibited a high concentration of Co^3+^ species (70.1%) compared with 45.3% of NiCo_2_S_4_ sample. Mixed oxidation states of Co were related to the presence of various sulfur oxidation states (S^2−^, S_2_^2−^, C–S) as observed in Fig. 6c for CuCo_2_S_4_ and (d) for NiCo_2_S_4_. The NiCo_2_S_4_ sample in Fig. 6d also showed the presence of 10.3% of C–S at 165.1 eV from metal ethyl xanthate precursor compounds and 17.7% sulfate (SO_4_^2−^) peak at 168.4 eV as a result of high-temperature treatment. Fig. 6e shows Cu 2p with different oxidation states at 932.3 eV for Cu^0^ and 934.1 and 935.8 eV for Cu^2+^ oxidation states. The corresponding spin-spit coupling components (Δ*E* = 19.4 eV) were observed at high binding energies. The Ni 2p peak for NiCo_2_S_4_ sample in Fig. 6f also showed mixed oxidation states at different binding energies with 26.4% metallic (Ni^0^) at 853.2 eV and Ni^2+^ at 855.9 (51.2%) and 858.5 eV (21.4%).

### 3.2. Electrochemical studies

Henceforth, the NiCo_2_S_4_ nanoparticles synthesized at 200 °C and 300 °C will be referred to as NCS-200 and NCS-300, respectively, whereas CuCo_2_S_4_ nanoparticles synthesized at 200 °C and 300 °C will be referred to as CCS-200 and CCS-300 respectively.

#### 3.2.1. Energy storage application

**Fig. 7:**
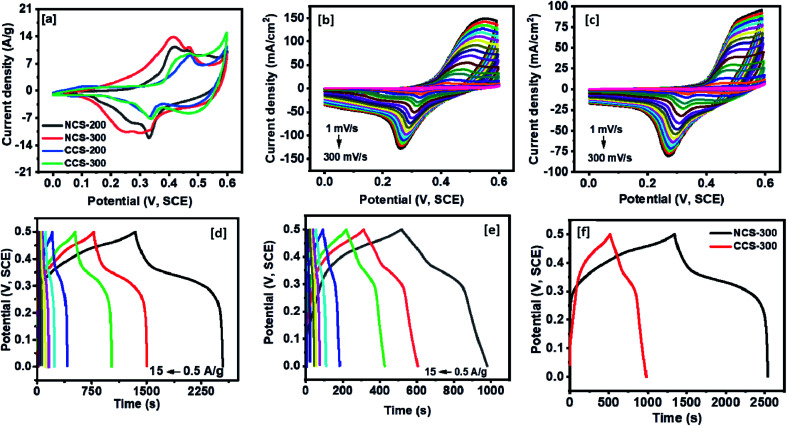
(a) Comparison data for CV curves at the scan rate of 5 mV s^−1^ for all the NCS and CCS samples. CV curves at various scan rates for NCS-300 (b) and (c) CCS-300. The potential *vs.* time plots at various current densities for (d) NCS-300 and (e) CCS-300. (f) CD curves for NCS-300 and CCS-300 at the current density of 0.5 A g^−1^.

The electrochemical charge storage mechanism of the synthesized NiCo_2_S_4_ and CuCo_2_S_4_ at different annealing temperatures was first analyzed using CV tests. As observed from the CV curves at 5 mV s^−1^, all the samples showed a distinct set of oxidation and reduction peaks (Fig. 7a). More specifically, a higher intensity of redox peak indicates improved intercalation of electrolyte ions within the interstitial sites of the thiospinels structure.^37^ As predicted by this study, the 300 °C annealed samples showed a well-defined structure with minimal nanoparticle agglomeration, allowing a higher exposed surface area for the improved pseudocapacitive performance compared to the 200 °C annealed samples. The CV curves for NCS-300 and CCS-300 electrodes at varying scan rates can be summarized from Fig. 7b and c. As the scan rates were decreased from 300 mV s^−1^ to 1 mV s^−1^, pseudocapacitive characteristics of the synthesized electrodes were observed. This could be due to the improved accessibility of the electrolyte ions to facilitate near-surface redox reactions provided by our annealing process. The real-time charge storage performance of NCS-300 and CCS-300 was further analyzed using galvanostatic charge–discharge (CD) test in which a longer charge–discharge time was displayed by NCS-300, indicating higher charge storage activity (Fig. 7d–f).

**Fig. 8:**
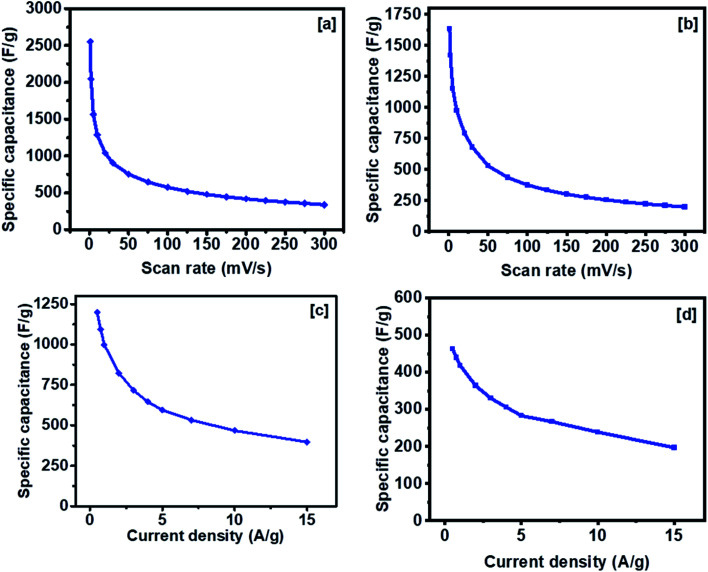
Variation of specific capacitance at different scan rates from 1 to 300 mV s^−1^ (a, b). Variation of specific capacitance *vs.* current density from 0.5 to 15 A g^−1^ (c, d).

The specific capacitance of NCS-300 and CCS-300 as a function of scan rates are shown in Fig. 8a and b. NCS-300 displayed the highest specific capacitance of *ca*. 2650 F g^−1^ at 1 mV s^−1^, whereas CCS-300 achieved a specific capacitance of *ca*. 1700 F g^−1^ at the same scan rate. The variation of specific capacitance of the two electrodes as a function of current density is shown in Fig. 8c and d. It can be seen that NCS-300 exhibits the highest specific capacitance of 1201 F g^−1^ at the applied current density of 0.5 A g^−1^. This value is much higher compared to several other reported NiCo_2_S_4_ electrodes prepared by other methods. For instance, Pu *et al.* employed a sacrificial template approach based on the Kirkendall effect to synthesize hollow hexagonal NiCo_2_S_4_ nanoplates, which delivered a specific capacitance of 437 F g^−1^ at a current density of 1 A g^−1^.^38^ Xu *et al.* synthesized NiCo_2_S_4_ nanoparticles *via* a one-step solvothermal approach, which displayed a specific capacitance of 519.51 F g^−1^ at a current density of 2 A g^−1^.^39^ Similarly, mesoporous NiCo_2_S_4_ nanosheets prepared by Wu *et al. via* a one-pot hydrothermal method displayed a specific capacitance of 744 F g^−1^ at a current density of 1 A g^−1^.^40^ A further comparison with composites or heterostructures of NiCo_2_S_4_ indicates that the specific capacitance observed for NCS-300 is also higher than,^41^ or comparable to^39^ several other values reported for NiCo_2_S_4_-based electrodes. As it is well-known, the synthetic methodology employed in the preparation of nanomaterials has a high influence on the properties and applications of the materials. It can therefore be speculated that the solventless approach employed herein plays an important on the observed performance. This is due to the fact that the method allows the synthesis of nanomaterials free of long-chained capping ligands,^42^ which have been shown to be detrimental to the electrochemical performance of the materials.^25–27^ Contrary to the performance observed for NCS-300, CCS-300 showcased a specific capacitance of 475 F g^−1^ at the same current density of 0.5 A g^−1^. Although this performance is much lower than that of NCS-300, it is still higher than^43^ or comparable to^38,44^ some of the previously reported CuCo_2_S_4_/NiCo_2_S_4_-based electrodes. Generally, NCS-300 displayed better energy storage characteristics than CCS-300, which can be attributed to its high conductivity.^45^ A detailed comparison of the observed specific capacitance values with previously reported values for unary and binary metal oxide/sulfide systems is given in Table S1.†

#### 3.2.2. Energy generation application

Considering the higher oxidation and reduction activity of the 300 °C annealed samples, we tried to further analyze their electrocatalytic performance on the overall water splitting reaction for the fuel cell applications.

**Fig. 9:**
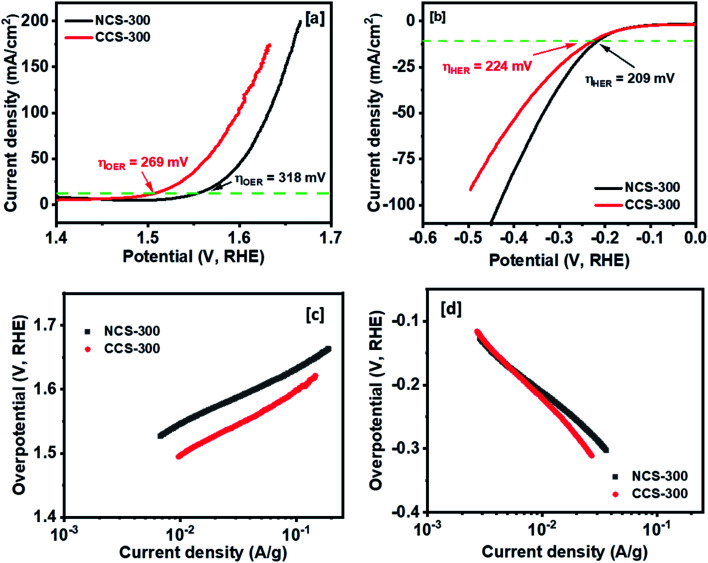
OER activity for NCS-300 and CCS-300. Polarization curves for OER activity (a) and HER activity (b) at a scan rate of 2 mV s^−1^. Tafel plots of OER (c) and HER (d) process for NCS-300 and CCS-300.

The OER and HER activity of the synthesized NCS-300 and CCS-300 electrodes can be summarized in Fig. 9. Unlike supercapacitors, the catalytic activity observed from the polarization curves for NCS-300 and CCS-300 shows a distinct behavior. This is due to two different sets of reaction mechanisms involved in HER and OER reactions. The overall water splitting mechanism includes adsorption and desorption of hydrogen and oxygen atoms by breaking the water molecule over the catalyst surface.^46^ The thiospinel structure facilitates tetrahedral and octahedral coordination to allow bi-functional oxygen and hydrogen evolution reactions.^46*b*^ Both the thiospinel catalysts showed high OER and HER activity. The CCS-300 showed higher OER catalysis with a low overpotential of 269 mV than NCS-300 (318 mV) at 10 mA cm^−2^ (Fig. 9a). On the other hand, NCS-300 showed higher HER catalysis with a low overpotential of 209 mV as compared to CCS-300 (224 mV) at 10 mA cm^−2^ (Fig. 9b). The high OER/HER activity could be due to coordination between high spin Co^3+^ and Cu^2+^/Ni^2+^ oxidation states with sulfur facilitating higher electronic conductivity for bifunctional catalysis.^47^ The NiCo_2_S_4_ nanowires/Ni foam electrode prepared by Sivanantham *et al.* achieved low overpotentials of 210 mV for HER and 260 mV for OER at 10 mA cm^−2^, which is comparable to CCS-300.^48^ The catalytic activity observed for CCS-300 is also comparable to that of CuCo_2_S_4_/NiCo_2_S_4_ electrocatalyst synthesized *via* a hydrothermal approach.^49^ Furthermore, the OER activity displayed by CCS-300 is superior to that of CuCo_2_S_4_ nanoparticles (395 mV at 10 mA cm^−2^),^50^ NiCo_2_S_4_@N/S-rGO composite (470 mV at 10 mA cm^−2^),^51^ and CuCo_2_S_4_ nanosheets (310 mV at 10 mA cm^−2^),^52^ prepared by other methods. A more detailed comparison for the HER/OER activity of CCS-300 and NCS-300 with previously reported catalysts is summarized in Table S2.† The Tafel plots for HER/OER activity for the two catalysts are summarized in Fig. 9c and d. The kinetics of the catalytic reaction observed from the Tafel plots directly correlate to the HER/OER performance observed during the polarization test in Fig. 9a and b. The Tafel slopes for the OER activity were calculated to be 89 and 103 mV dec^−1^ for NCS-300 and CCS-300, respectively, whereas the Tafel slopes for the HER activity were found to be 150 and 185 mV dec^−1^ for NCS-300 and CCS-300, respectively. For comparison purposes, the electrochemical performance of commercial Pt catalyst was also measured. As seen in Fig. S3,† Pt is a good catalyst for the HER process (overpotential of 33 mV); however, the catalyst displays an overpotential of 545 mV for the OER process, which is higher than that displayed by CCS-300 (269 mV).

**Fig. 10:**
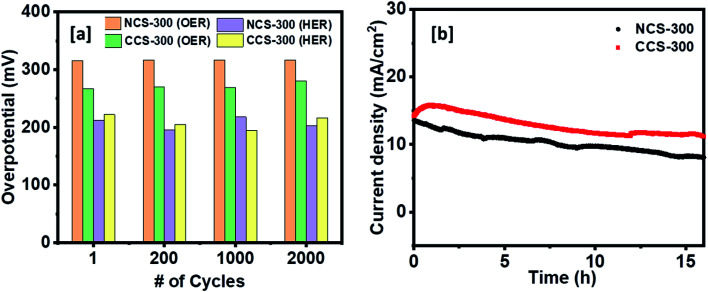
(a) The overpotential values at 10 mA cm^−2^ for HER and OER reactions for stability test for 2000 cycles. (b) Chronoamperometry stability test for NCS-300 and CCS-300.

The electrocatalytic stability test for both the catalyst electrodes was further examined for 2000 cycles. As shown in Fig. 10a, both the catalysts showed a negligible change in their OER/HER catalytic performance and maintained their low overpotential values for 2000 cycles. The OER chronoamperometry curves in Fig. 10b shows that both the catalysts retained their high current value corresponding to stable catalytic activity after continuous polarization for more than 15 h.

**Fig. 11:**
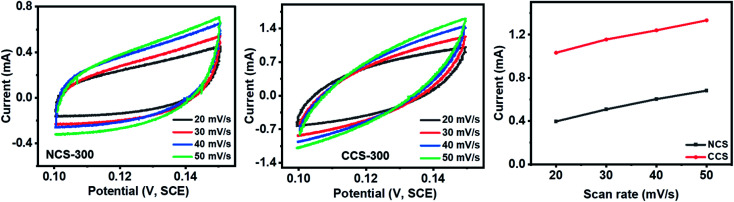
CV curves at various scan rates for NCS-300 and CCS-300.

The electrochemical surface area of NCS-300 and CCS-300 was calculated by performing CV at various scan rates in non-faradic regions. The plotting relation of current density with scan rate is found to be linear. The slope was observed to be 38 mC cm^−2^ for NCS-300 and 39.4 mC cm^−2^ for CCS-300. The higher slope (electrochemical surface area) of CCS-300 suggests better electrochemical properties for the water-splitting application (Fig. 11).

## 4. Conclusion

In summary, we have employed a facile, solventless approach to synthesize uncapped NiCo_2_S_4_ and CuCo_2_S_4_ nanoparticles for supercapacitance and water splitting applications. The synthesis was achieved *via* thermolysis of respective metal ethyl xanthate precursors at 200 and 300 °C. p-XRD and TEM analyses reveal the synthesis of highly crystalline phase pure nanomaterials with some degree of particle–particle agglomeration. Electrochemical characterizations indicate that the particles synthesized at a high temperature of 300 °C exhibits better energy storage characteristics. The highest specific capacitance of 1201 F g^−1^ at a current density of 0.5 A g^−1^ was observed for NiCo_2_S_4_ nanoparticles, whereas CuCo_2_S_4_ nanoparticles displayed a specific capacitance of 475 F g^−1^ at the same current density. Conversely, the best electrocatalytic activity was observed for CuCo_2_S_4_ nanoparticles, which require overpotentials of 269 mV for OER, and 224 mV for HER, to deliver a current density of 10 mA cm^−2^. The stability of the catalysts was examined for 2000 cycles in which a minor change in both OER and HER activities was observed.

## Conflicts of interest

There are no conflicts to declare.

## Supplementary Material

RA-011-D1RA02309H-s001
