# Supplementary material for: Low temperature scalable synthetic approach enabling high bifunctional electrocatalytic performance of NiCo2S4 and CuCo2S4 thiospinels
Source: RSC Adv. 2021 Sep 24;11(50):31533–46. doi: 10.1039/d1ra02309h (PMC9041439; doi:10.1039/d1ra02309h)
Supplement: RA-011-D1RA02309H-s001 [file RA-011-D1RA02309H-s001.pdf]

Electronic Supplementary Material (ESI) for RSC Advances.
This journal is © The Royal Society of Chemistry 2021

# Supplementary Information

# Low temperature scalable synthetic approach enabling high bifunctional electrocatalytic performance of $NiCo_2S_4$ and $CuCo_2S_4$ thiospinels

Ginena Bildard Shombe,[1,2] Shumaila Razzaque,[3] Malik Dilshad Khan,[1,4]* Tebello Nyokong,[5] Philani Mashazi,[5,6] Jonghyun Choi,[7] Sanket Bhoyate,[7] Ram K. Gupta,[7] and Neerish Revaprasadu[1]*

[1]Department of Chemistry, University of Zululand, Private Bag X1001, KwaDlangezwa 3880, South Africa.
[2]Chemistry Department, University of Dar-es-Salaam, P.O. Box 35061, Dar-es-Salaam, Tanzania.
[3]Key Laboratory of Material Chemistry for Energy Conversion and Storage, Ministry of Education, Hubei Key Laboratory of Material Chemistry and Service Failure, School of Chemistry and Chemical Engineering, Huazhong University of Science and Technology, Luoyu Road No. 1037, Wuhan, China.
[4]Institute of Physical Chemistry, Polish Academy of Sciences, Kasprzaka 44/52, 01-224 Warsaw, Poland.
[5]Institute for Nanotechnology Innovation, P.O. Box 94, Rhodes University, Makhanda 6140, South Africa.
[6]Department of Chemistry, P. O. Box 94, Rhodes University, Makhanda 6140, South Africa.
[7]Department of Chemistry, Pittsburg State University, Pittsburg, KS 66762, USA.

*Email: RevaprasaduN@unizulu.ac.za; malikdilshad@hotmail.com

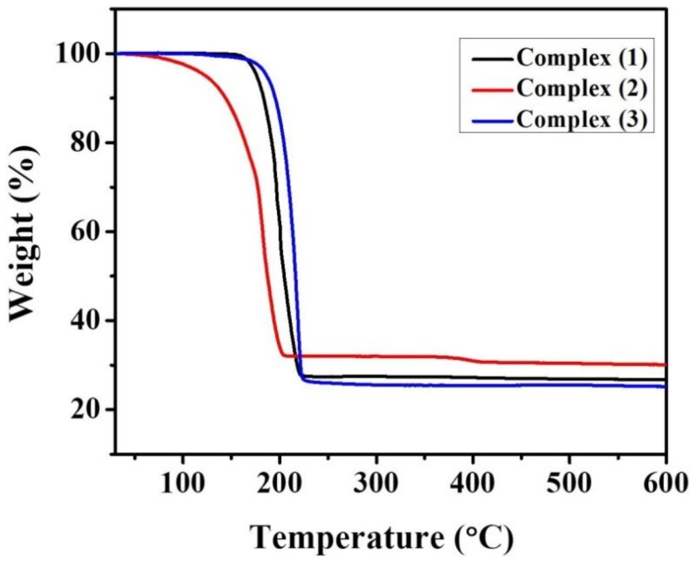


**Figure S1.** TGA curves of complexes nickel ethyl xanthate (**1**), copper ethyl xanthate (**2**), and cobalt ethyl xanthate (**3**).

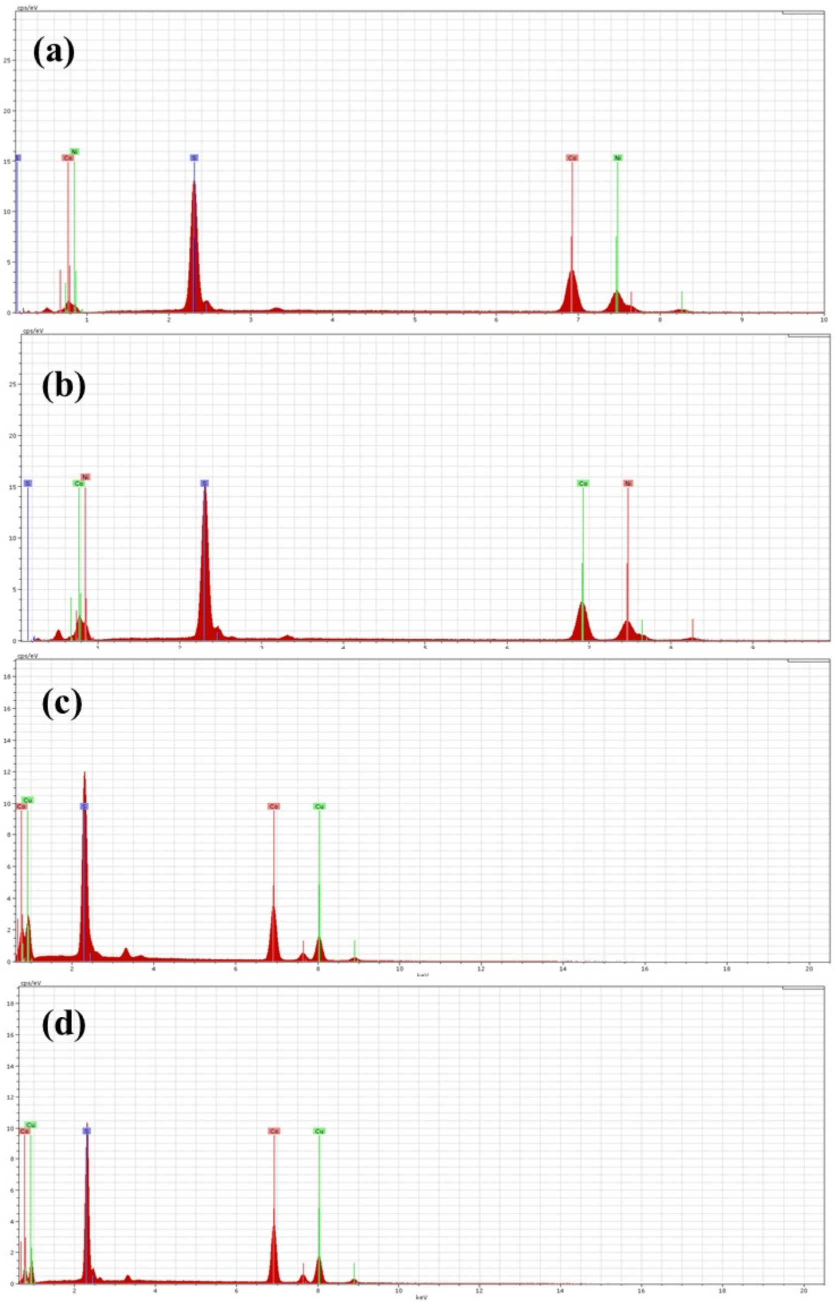


**Figure S2.** EDX spectra of $NiCo_2S_4$ synthesized at (a) 200 $^oC$ and (b) 300 $^oC$. EDX spectra of $CuCo_2S_4$ synthesized at (c) 200 $^oC$ and (d) 300 $^oC$

**Table S1**. Comparison chart with previously reported supercapacitor electrodes.

| Electrode Material | Synthetic Method | Specific Capacitance (F/g) | Current Density (A/g) | Reference |
|---|---|---|---|---|
| $NiCo_2S_4$ | Sacrificial template method | 437 | 1 | 1 |
| $NiCo_2S_4$ | Solvothermal | 519.51 | 2 | 2 |
| $NiCo_2S_4$ | Hydrothermal | 744 | 1 | 3 |
| $NiCo_2S_4$@$MnO_2$ | Hydrothermal | 520.7 | 1 | 4 |
| AMC@ $NiCo_2S_4$ | Hydrothermal | 651.1 | 0.6 | 5 |
| $CuCo_2S_4$/polyacrylonitrile | | 385 | 1 | 6 |
| $CuCo_2S_4$@$NiCo_2S_4$ | Hydrothermal | 539.2 C/g | 1 | 7 |
| $CuCo_2S_4$ | Hydrothermal and sulfuration process | 373.4 | 1 | 8 |
| $CuCo_2S_4$ | Anion-exchange and post-annealing | 424 | 1 | 9 |
| $CuCo_2S_4$/Graphene | Hydrothermal | 525.4 | 1 | 10 |
| $CuCo_2S_4$ | Two-step hydrothermal approach | ~300 | 0.17 (mA/cm$^2$) | 11 |
| $CuCo_2O_4$ | Hydrothermal | 285.5 | 0.5 | 12 |
| $Co_3O_4$ | Hydrothermal | 272.86 | 0.5 | 13 |
| $NiCo_2O_4$ | Hydrothermal | 225 | 1 | 14 |
| $CoFe_2O_4$ | Electrodeposition | 768 | 0.5 | 15 |
| $CuCo_2S_4$ | hydrothermal and sulfuration process | 373.4 | 1 | 8 |
| $AgBiS_2$ | Melt method | 175 | 0.5 | 16 |
| **$CuCo_2S_4$ (CCS-300)** | **Solventless approach** | **475** | **0.5** | **This work** |
| **$NiCo_2S_4$ (NCS-300)** | **Solventless approach** | **1200** | **0.5** | |

*AMC-Activated multiporous carbon

**Table S2**. Comparison chart with previously reported electrocatalyst electrodes.

| Catalyst Materials | Synthetic Method | $\eta_{HER}$ (mV) at 10 mA/cm$^2$ | $\eta_{OER}$ (mV) at 10 mA/cm$^2$ | Reference |
|---|---|---|---|---|
| $CuCo_2S_4$/$NiCo_2S_4$ | Hydrothermal | 206 | 271 | 17 |
| $CuCo_2S_4$ | Hydrothermal | - | 310 | 18 |
| $CuCo_2S_4$ | Hydrothermal | 158 | 290 (20 mA/cm$^2$) | 12 |
| $NiCo_2O_4$ | Hydrothermal | - | 346 | 19 |
| $NiCo_2S_4$ | Solvothermal | 226 | - | 20 |
| $NiCo_2S_4$ | Hydrothermal followed by sulfidation | - | 309 | 19 |
| $NiCo_2O_4$/NF | Solvothermal | ~250 | ~350 | 21 |

| | | | | |
|---|---|---|---|---|
| $NiCo_2Se_4$ | Hydrothermal | | 270 | 19 |
| $NiCo_2S_4$/NF | Solvothermal | ~200 | ~330 | 21 |
| $Ni_3S_2$/NF | Hydrothermal and thermal sulfurization process | 271 | - | 22 |
| $NiCo_{-LDH}$/NF | Hydrothermal | 231 | - | 22 |
| $NiCo_2S_4$/NF | Hydrothermal | 210 | 260 | 23 |
| $NiCo_2S_4$/NF | Two-step hydrothermal method | 191 | <251 (40 mA/cm$^2$) | 24 |
| $NiCo_2S_4$@N/S-rGO | Solvothermal | - | 470 | 25 |
| $NiCo_2O_4$ NS/CC | Hydrothermal | - | 368 | 26 |
| $NiCo_2S_4$ NS/CC | Hydrothermal | - | 316 | 26 |
| $CuCo_2S_4$ | Solution-based chemical route | - | 395 | 27 |
| FeO@$CuCo_2S_4$ | Hydrothermal | - | 240 | 28 |
| **CCS-300** | **Solventless approach** | **224** | **269** | **This Work** |
| **NCS-300** | **Solventless approach** | **209** | **318** | |

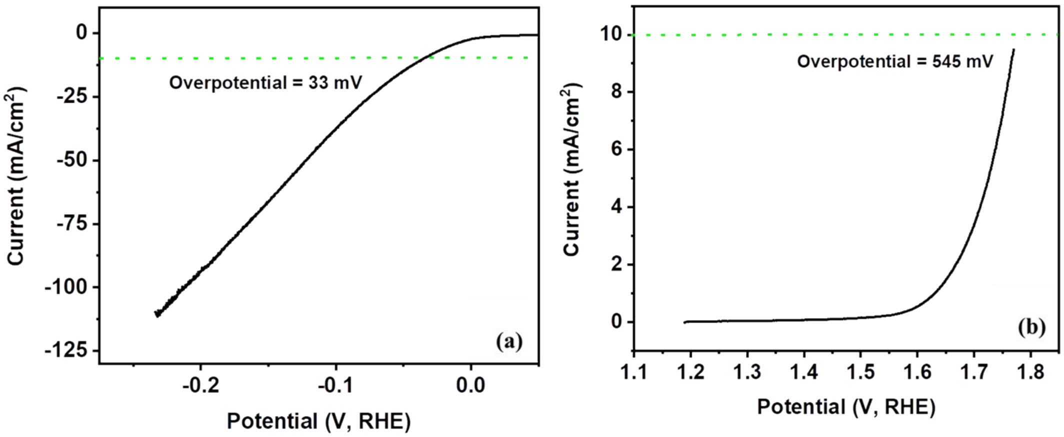


**Figure S3.** Electrocatalytic activity of commercial platinum for HER (a) and OER (b) processes.

## References


1 Pu, J. *et al.* Preparation and electrochemical characterization of hollow hexagonal $NiCo_2S_4$ nanoplates as pseudocapacitor materials. *ACS Sustainable Chemistry & Engineering* **2**, 809-815 (2014).

2 Xu, J. *et al.* Novel NiCo$_2$S4@ reduced graphene oxide@ carbon nanotube nanocomposites for high performance supercapacitors. *RSC Advances* **6**, 100504-100510 (2016).

3 Wu, Z. *et al.* High energy density asymmetric supercapacitors from mesoporous $NiCo_2S_4$ nanosheets. *Electrochimica Acta* **174**, 238-245 (2015).

4 Chen, H., Liu, X. L., Zhang, J. M., Dong, F. & Zhang, Y. X. Rational synthesis of hybrid $NiCo_2S_4$@ $MnO_2$ heterostructures for supercapacitor electrodes. *Ceramics International* **42**, 8909-8914 (2016).

5 Wang, T. *et al.* Facile preparation and sulfidation analysis for activated multiporous carbon@ $NiCo_2S_4$ nanostructure with enhanced supercapacitive properties. *Electrochimica Acta* **211**, 627-635 (2016).

6 Chen, L., Zuo, Y., Zhang, Y. & Gao, Y. A novel $CuCo_2S_4$/polyacrylonitrile ink for flexible film supercapacitors. *Materials Letters* **215**, 268-271 (2018).

7 Ma, L., Chen, T., Li, S., Gui, P. & Fang, G. A 3D self-supported coralline-like $CuCo_2S_4$@ $NiCo_2S_4$ core-shell nanostructure composite for high-performance solid-state asymmetrical supercapacitors. *Nanotechnology* **30**, 255603 (2019).

8 Li, H. *et al.* Enhanced electrochemical performance of $CuCo_2S_4$/carbon nanotubes composite as electrode material for supercapacitors. *Journal of colloid and interface science* **549**, 105-113 (2019).

9 Cheng, J. *et al.* Influence of crystallinity of $CuCo_2S_4$ on its supercapacitive behavior. *Journal of Alloys and Compounds* **825**, 153984 (2020).

10 Annamalai, K. & Tao, Y.-s. A hierarchically porous $CuCo_2S_4$/graphene composite as an electrode material for supercapacitors. *New Carbon Materials* **31**, 336-342 (2016).

11 Wang, Q., Liang, X., Yang, D. & Zhang, D. Facile synthesis of novel $CuCo_2S_4$ nanospheres for coaxial fiber supercapacitors. *RSC advances* **7**, 29933-29937 (2017).

12 Zequine, C. *et al.* Effect of solvent for tailoring the nanomorphology of multinary $CuCo_2S_4$ for overall water splitting and energy storage. *Journal of Alloys and Compounds* **784**, 1-7 (2019).

13 Adhikari, H. *et al.* Synthesis and electrochemical performance of hydrothermally synthesized $Co_3O_4$ nanostructured particles in presence of urea. *Journal of Alloys and Compounds* **708**, 628-638 (2017).

14 Ghimire, M. *et al.* Physical Properties and Theoretical Study of $NixCo_{3-x}O_4$ ($0\leq x\leq 1.5$) Nanostructures as High-Performance Electrode Materials for Supercapacitors. *Journal of nanoscience and nanotechnology* **19**, 4481-4494 (2019).

15 Zhang, C. *et al.* Electrodeposited nanostructured $CoFe_2O_4$ for overall water splitting and supercapacitor applications. *Catalysts* **9**, 176 (2019).

16 Khan, M. D. *et al.* Electrochemical investigation of uncapped $AgBiS_2$ (schapbachite) synthesized using in situ melts of xanthate precursors. *Dalton Transactions* **48**, 3714-3722 (2019).

17 Ma, L. *et al.* 3D $CuCo_2S_4$/$NiCo_2S_4$ core-shell composites as efficient bifunctional electrocatalyst electrodes for overall water splitting. *Electrochimica Acta* **326**, 135002 (2019).

18 Chauhan, M., Reddy, K. P., Gopinath, C. S. & Deka, S. Copper cobalt sulfide nanosheets realizing a promising electrocatalytic oxygen evolution reaction. *ACS Catalysis* **7**, 5871-5879 (2017).

19 Jeghan, S. M. N. & Lee, G. One-dimensional hierarchical nanostructures of $NiCo_2O_4$, $NiCo_2S_4$ and $NiCo_2Se_4$ with superior electrocatalytic activities toward efficient oxygen evolution reaction. *Nanotechnology* (2020).

20 Sheng, G. *et al.* Flowerlike $NiCo_2S_4$ hollow sub-microspheres with mesoporous nanoshells support Pd nanoparticles for enhanced hydrogen evolution reaction electrocatalysis in both acidic and alkaline conditions. *ACS applied materials & interfaces* **10**, 22248-22256 (2018).

21 Yin, X. *et al.* 3D hierarchical network $NiCo_2S_4$ nanoflakes grown on Ni foam as efficient bifunctional electrocatalysts for both hydrogen and oxygen evolution reaction in alkaline solution. *International Journal of Hydrogen Energy* **42**, 25267-25276 (2017).

22 Liu, H. *et al.* Heteromorphic $NiCo_2S_4/Ni_3S_2$/Ni foam as a self-standing electrode for hydrogen evolution reaction in alkaline solution. *ACS applied materials & interfaces* **10**, 10890-10897 (2018).

23 Sivanantham, A., Ganesan, P. & Shanmugam, S. Hierarchical $NiCo_2S_4$ nanowire arrays supported on Ni foam: an efficient and durable bifunctional electrocatalyst for oxygen and hydrogen evolution reactions. *Advanced Functional Materials* **26**, 4661-4672 (2016).

24 Gong, Y. *et al.* Crossed $NiCo_2S_4$ Nanowires Supported on Nickel Foam as a Bifunctional Catalyst for Efficient Overall Water Splitting. *ChemistrySelect* **4**, 1180-1187 (2019).

25 Liu, Q., Jin, J. & Zhang, J. $NiCo_2S_4$@ graphene as a bifunctional electrocatalyst for oxygen reduction and evolution reactions. *ACS applied materials & interfaces* **5**, 5002-5008 (2013).

26 Hyun, S. & Shanmugam, S. Hierarchical Nickel–Cobalt Dichalcogenide Nanostructure as an Efficient Electrocatalyst for Oxygen Evolution Reaction and a Zn–Air Battery. *ACS omega* **3**, 8621-8630 (2018).

27 Wiltrout, A. M., Read, C. G., Spencer, E. M. & Schaak, R. E. Solution synthesis of thiospinel CuCo2S4 nanoparticles. *Inorganic chemistry* **55**, 221-226 (2016).

28 Ahmed, A. T. A., Pawar, S. M., Inamdar, A. I., Im, H. & Kim, H. Fabrication of FeO@ $CuCo_2S_4$ multifunctional electrode for ultrahigh-capacity supercapacitors and efficient oxygen evolution reaction. *International Journal of Energy Research* **44**, 1798-1811 (2020).
